# Modified safe technique for circumcision

**DOI:** 10.4103/0970-0358.41110

**Published:** 2008

**Authors:** Nitin Mokal, Navdeep Chavan

**Affiliations:** Department of Plastic Surgery, Grant Medical College, GT Hospital, LT Marg, Dhobitalao, Mumbai - 400 001, Maharashtra, India

**Keywords:** Circumcision, packing gauze (tape gauze)

## Abstract

We have used surgical gauze under the prepuceal skin as a pack in 20 cases prior to marking incision for circumcision. The prepuceal adhesions were first dissected and seperated. The method allows a stable, well-supported prepuceal surface for marking incisions and avoids injuries to the glans. Because the prepuceal surface is taut and stable, hemostasis is easier and quicker and the operating time is reduced.

## INTRODUCTION

Around 1/6^th^ of the world's male population is reported to have been circumcised.[1] Nearly 1.2 million newborn males are circumcised yearly in the USA and nearly 30,000 in the UK.[2,3] Around 33% of the general population in India is circumcised.[4] The earliest Egyptian mummies were circumcised in 1300 B.C. This is a procedure done by various specialists and yet the results have been good with an overall complication rate of 0.2-6%.[5–7] Hemorrhage and infection are the most common complications followed by wound dehiscence, recurrent phimosis, prepuceal adhesions, trauma to the glans and an ugly scar. In our study, we have practised the use of packing gauze in between the prepuce and the glans in a formal circumcision. This method allows meticulous homeostasis, prevents trauma to the glans and gives good support for cutting the foreskin. The operative time is shorter.

## MATERIALS AND METHODS

Twenty healthy young males aged 2-25 years were operated in the period from January 2004 to December 2004 in our Plastic surgery department. There were “no exclusion criteria”. The indications for surgery were congenital or acquired phimosis, recurrent balanoposthitis and religious reasons.

**Figure 1:**
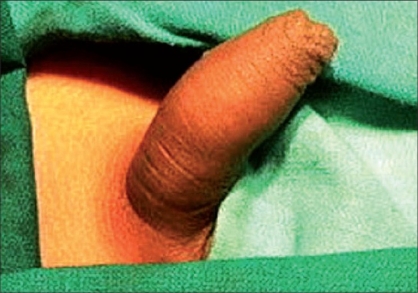
Preoperative photo

**Figure 2:**
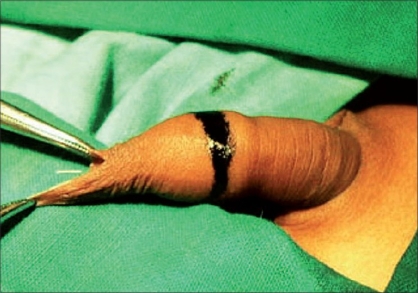
Coronal marking

**Figure 3:**
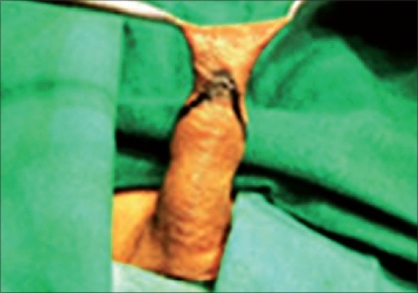
Ventral marking

**Figure 4:**
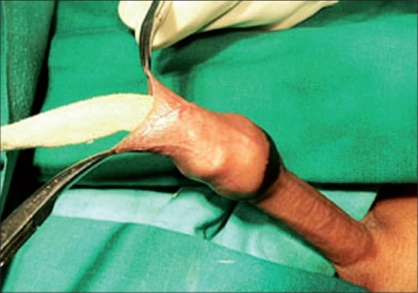
Packing gauge

**Figure 5:**
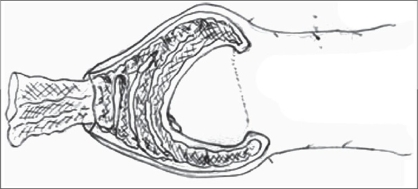
Packing gauge

Routine blood and urine tests were performed and consent for surgery was taken. The operation was performed under local anesthesia (dorsal penile nerve block) in the older patients while general anesthesia with caudal epidural block was employed in young children. In our study, we broke adhesions with the help of a small artery forceps when the patient was under the effect of anesthesia. Marking was done on the outer skin at the level of the coronal sulcus tapering towards the frenulum, packing gauze was filled in between the prepuce and the glans and an incision was made on the outer layer of the skin [Figures 1-5].

**Figure 6:**
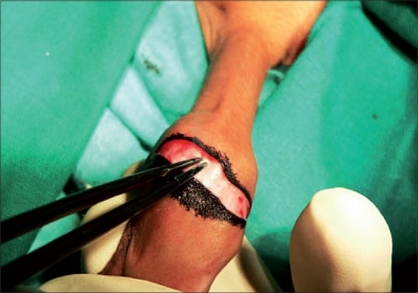
Coagulation of dorsal vein

**Figure 7:**
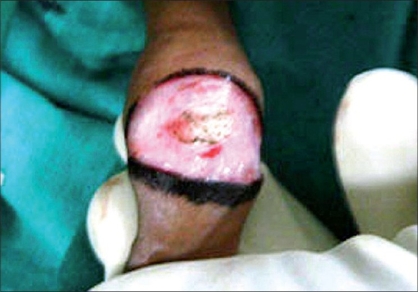
Cut both layers of prepuce

**Figure 8:**
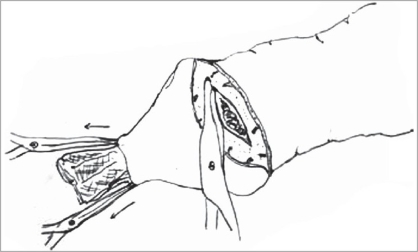
Extension of inner layer cut

**Figure 9:**
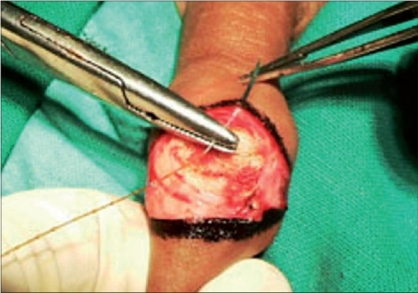
Closure of both layers

**Figure 10:**
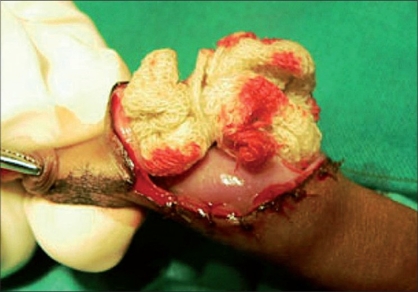
Closure complete

**Figure 11:**
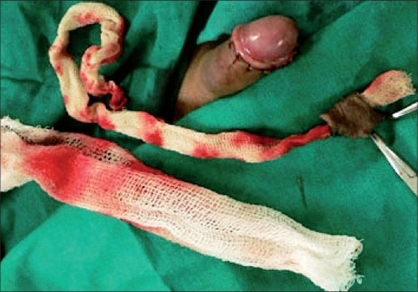
Cuff of excised prepuce and blood loss

**Figure 12:**
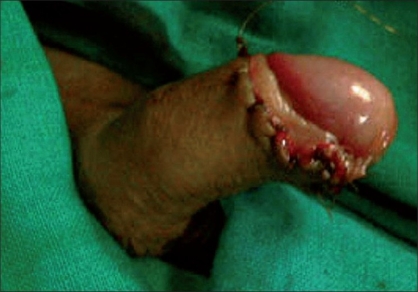
Final closure

The packing gauze gives support while making the incision and also exposes the dorsal vessels which can be easily coagulated with a bipolar cautery [Figures 6-8]. An incision was made on the inner layer of the prepuceal skin which was stretched because of the packed gauze between the glans and the inner layer of the prepuce. The edges of the outer and inner layers were approximated with 5-0 chromic catgut and the incision was completed by taking care of the frenular artery with a figure of “8” suture. In this technique, all the blood vessels were coagulated before cutting the inner prepuceal layer so the blood loss was minimal [Figures 9-12].

A local antibiotic cream was applied along the suture line. All the patients received a course of antibiotic (amoxicillin and cloxacillin combination) and analgesics for seven days with a local antibiotic cream application along suture line. Patients were allowed to take bath after the 3^rd^ postoperative day. All patients were admitted as “day care surgery” and followed up in the outpatient department for three months.

## RESULTS

The ages of the patients ranged from 2 to 25 years.

Indication: Congenital Phimosis - 9

Religious - 6

Recurrent balanoposthitis - 3

Acquired phimosis - 2

The average time taken for the procedure was 15 min (10-20 min). The average healing period was between 7-10 days with a mean of 8 days.

### Complications

No major complication was seen except edema around the proximal portion of the penis which settled in 8-12 days.

## DISCUSSION

There are many techniques of circumcision described in literature. They are classified in different ways but broadly classified as dorsal slit technique, clamp, sleeve excision, use of shield and sutureless technique.[8] All these techniques have their pros and cons and some of them are not in use nowadays. Some of the contraptions used in the techniques are outdated. The ultimate aim of these numerous techniques is to decrease complications and reduce time taken to operate and improve cosmesis.

In our study, we have marked the line of incision on the outer layer of the prepuceal skin to prevent inadvertent excision of excess prepucial skin. We have used packing gauze to fill the space between the prepuce and the glans to give protection to the glans and provide a stable and relatively rigid base while taking the incision. The packing also causes traction on the dorsal vessels which can be easily identified and coagulated with bipolar cautery after the outer skin incision is taken.

Subsequently the inner layer was incised, at this stage, these vessels get retracted but are already coagulated in this technique unlike in a standard technique where they get retracted in bleeding conditions and we may have to depend on deep hemostatic sutures which causes more tissue trauma and delayed healing. On the ventral aspect, frenular artery coagulation is achieved and is followed by a figure of “8” suture. Suturing of both layers of the prepuce was done with 5-0 chromic catgut.

## CONCLUSION

**Figure 13:**
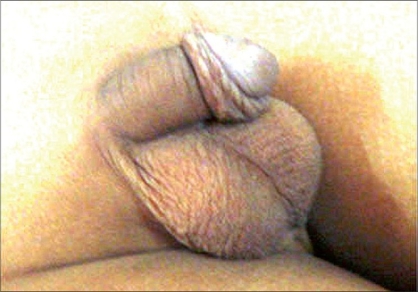
Long-term result

This procedure was less time-consuming and allowed more efficient hemostasis. There were no major complications in our study. As such, there were no drawbacks to this technique and it is safe in less experienced hands. In terms of cost-effectiveness, this technique was cheap and can be done in a small setup without the need of specialized instruments. This technique provides better aesthetic results [Figure 13]. Thus, it can be considered as a modified safe technique for circumcision.

## References

[1] Waszak SJ (1978). The historic significance of circumcision. Obstet Gynaecol.

[2] Lazim TR, Zainol J (1996). A Simple device for circumcision. JR Coll Surg Edinb.

[3] Zafar F, Thompson JN, Pati J, Abed PD (1993). Suture less circumcision. Br J Surg.

[4] Reynolds SJ, Shepherd ME, Risbud AR, Gangakhedkar RR, Brookmeyer RS, Divekar AD (2004). Male circumcision and risk of HIV-1 and other sexually transmitted infections in India. Lancet.

[5] Williams N, Kapilla L (1993). Complication of circumcision. Br J Surg.

[6] Horowtz M, Gershbein AB (2001). Gomco circumcision: When it is safe?. J Pediatr Surg.

[7] Wakefield SE, Elewa AA (1995). Adult circumcision under local anaesthetic. Br J Urol.

[8] Sharma PP (2004). Sutureless circumcision: Wound closure after circumcision with cynoacrylate glue - A preliminary Indian study. Indian J Surg.

